# Exploring simvastatin, an antihyperlipidemic drug, as a potential topical antibacterial agent

**DOI:** 10.1038/srep16407

**Published:** 2015-11-10

**Authors:** Shankar Thangamani, Haroon Mohammad, Mostafa F. N. Abushahba, Maha I. Hamed, Tiago J. P. Sobreira, Victoria E. Hedrick, Lake N. Paul, Mohamed N. Seleem

**Affiliations:** 1 Department of Comparative Pathobiology, Purdue University College of Veterinary Medicine, West Lafayette, IN, USA; 2 Faculty of Veterinary Medicine, Assiut University, Assiut, Egypt; 3 Bindley Bioscience Center, Purdue University, West Lafayette, IN, USA

## Abstract

The rapid rise of bacterial resistance to traditional antibiotics combined with the decline in discovery of novel antibacterial agents has created a global public health crisis. Repurposing existing drugs presents an alternative strategy to potentially expedite the discovery of new antimicrobial drugs. The present study demonstrates that simvastatin, an antihyperlipidemic drug exhibited broad-spectrum antibacterial activity against important Gram-positive (including methicillin-resistant *Staphylococcus aureus* (MRSA)) and Gram-negative pathogens (once the barrier imposed by the outer membrane was permeabilized). Proteomics and macromolecular synthesis analyses revealed that simvastatin inhibits multiple biosynthetic pathways and cellular processes in bacteria, including selective interference of bacterial protein synthesis. This property appears to assist in simvastatin’s ability to suppress production of key MRSA toxins (α-hemolysin and Panton-Valentine leucocidin) that impair healing of infected skin wounds. A murine MRSA skin infection experiment confirmed that simvastatin significantly reduces the bacterial burden and inflammatory cytokines in the infected wounds. Additionally, simvastatin exhibits excellent anti-biofilm activity against established staphylococcal biofilms and demonstrates the ability to be combined with topical antimicrobials currently used to treat MRSA skin infections. Collectively the present study lays the foundation for further investigation of repurposing simvastatin as a topical antibacterial agent to treat skin infections.

The blockbuster statin drugs have revolutionized the treatment of cardiovascular disease, primarily by reducing low-density lipoprotein cholesterol (LDL-C) levels, leading to a decline in the morbidity and mortality associated with coronary artery diseases^1^. All statins drugs exert their effect by inhibiting the enzyme class I 3-hydroxy-3-methyl-glutaryl-CoenzymeA reductase (HMG-CoA) leading to decreased synthesis of cholesterol and increased removal of low-density lipoprotein (LDL) circulating in the body^2^,^3^. These drugs possess a good safety profile with limited side effects thus permitting their frequent use in reducing lipid levels in patients with high cholesterol levels.

In addition to their lipid-lowering effect, statins have been found to have potential use for other applications including influencing the host immune response via the drugs’ anti-inflammatory and immune-modulatory properties^4^. Furthermore, multiple reports have investigated the potential role of statins in preventing and treating various infectious diseases and have demonstrated that statins can prevent the establishment of infections (by decreasing host cholesterol synthesis^5^,^6^ limiting certain bacterial species’ ability to invade host cells) and potentially decrease the mortality rate attributed to bacterial infection^7^,^8^. Interestingly, several studies have shown that certain statins possess antimicrobial activity directly inhibiting growth of *Staphylococcus*, *Streptococcus*, *Enterococcus* and *Moraxella* spp^9^,^10^,^11^. In addition, simvastatin and atorvastatin are capable of increasing the mycobactericidal effect of rifampicin^12^. However, limited information is available regarding the mechanism by which statins exert their antibacterial effect, statins’ antimicrobial effect on Gram-negative pathogens, and potential applications for statins as novel antibacterial agents.

Given the tremendous pressure bacterial resistance to currently available antibiotics has placed on the healthcare system (with certain bacterial strains of *Staphylococcus aureus* and *Pseudomonas aeruginosa* exhibiting resistance to nearly every class of antibiotics), new antimicrobials are urgently needed to counter this significant public health challenge^13^,^14^. Repurposing existing drugs (initially approved for treatment of one clinical indication such as lowering cholesterol levels) that also possess antibacterial activity has the potential to expedite the process to discovering new antibacterial agents (given much of the rigorous safety, pharmacokinetic, and pharmacodynamic studies have already been conducted)^15^,^16^,^17^. Based upon preliminary studies performed to date, statins, in particular simvastatin, have potential to be repurposed as novel antibacterial agents. However additional research is required to understand statins’ antibacterial spectrum of activity, their antibacterial mechanism of action, and to elucidate potential clinical applications in the management of bacterial infections. In this study, we aim to lay the foundation for utilizing statins as topical antibacterial agents by investigating the antibacterial activity of statins and their spectrum of activity on clinically-relevant Gram-positive and Gram-negative pathogens, elucidating the antibacterial mode of action of the most active statin (simvastatin), examining the effect of simvastatin on specific virulence factors (such as bacterial toxins and disruption of staphylococcal biofilms) and finally to validate the therapeutic efficacy of simvastatin in an appropriate animal model of *S. aureus* infection. Our study reveals that simvastatin has considerable promise for use as a therapeutic agent to treat MRSA skin infections and does warrant further investigation as a novel topical antibacterial agent.

## Results

### *In vitro* antibacterial assays

#### (i) Screening statins for antibacterial activity

The antibacterial activity of eight statin drugs including simvastatin, atorvastatin, fluvastatin, lovastatin, mevastatin, pitavastatin, pravastatin and rosuvastatin were evaluated against two representative Gram-positive and Gram-negative bacterial pathogens (methicillin-resistant *Staphylococcus aureus* (MRSA) ATCC 43300 and *Pseudomonas aeruginosa* ATCC 15442 respectively) (see Supplementary Table S1 online). Simvastatin was the only drug capable of inhibiting MRSA ATCC 43300 growth with a minimum inhibitory concentration (MIC) value of 32 μg/ml. Interestingly, none of the statin drugs examined possessed antibacterial activity against *P. aeruginosa* ATCC 15442 (MIC > 1024 μg/ml), indicating simvastatin’s effectiveness as an antibacterial activity may be restricted to Gram-positive pathogens.

#### (ii) Activity of simvastatin against Gram-positive bacteria

Confirmation of simvastatin’s antibacterial activity against MRSA ATCC 43300 led us to examine simvastatin’s ability to inhibit growth of important multidrug-resistant strains of Gram-positive pathogens (Table 1 and see Supplementary Table S2,3). Simvastatin exhibited bacteriostatic activity against all methicillin-sensitive *S. aureus* (MSSA), MRSA, vancomycin-intermediate *S. aureus* (VISA), vancomycin-resistant *S. aureus* (VRSA), vancomycin-sensitive *Enterococcus*, vancomycin-resistant *Enterococcus* (VRE) and *Listeria monocytogenes* strains, inhibiting 90% of the strains (MIC_90_) tested at a concentration of 32 μg/ml. Simvastatin also inhibited growth of strains of *Streptococcus pneumoniae* and *Bacillus anthracis* with a MIC_90_ of 64 and 16 μg/ml respectively.

#### (iii) Activity of simvastatin against Gram-negative bacteria

The antimicrobial activity of simvastatin was next assessed against various Gram-negative pathogens (Table 2). Initial investigation indicated that simvastatin did not possess antibacterial activity against Gram-negative bacteria. However, when the outer membrane (OM) permeability in these bacteria was compromised using a sub-inhibitory concentration of colistin, simvastatin displayed antimicrobial activity against all tested strains of Gram-negative pathogens including *Acinetobacter baumannii*, *Escherichia coli, Salmonella Typhimurium, Klebsiella pneumoniae, and P. aeruginosa* with the MIC ranging from 8–32 μg/ml.

The antibacterial activity of simvastatin was further investigated against *E. coli* SM1411∆ *acrAB*, a strain that is deficient in the multidrug-resistant AcrAB efflux pump. Simvastatin alone was not active against *E. coli* SM1411∆ *acrAB* (MIC > 256 μg/ml). However simvastatin was able to inhibit growth of this strain when combined with colistin (the MIC was 16 μg/ml).

### Simvastatin inhibits multiple macromolecular synthesis pathways

**Figure 1:**
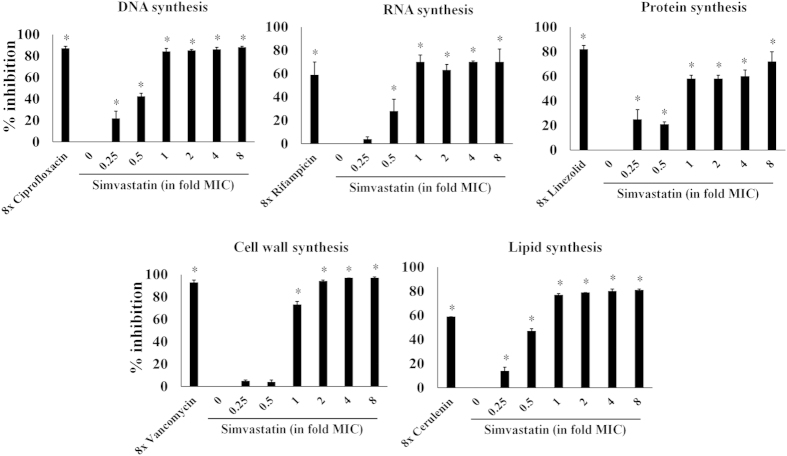
Macromolecular synthesis in the presence of simvastatin. Effect of simvastatin and control antimicrobials at indicated concentration (in fold MICs) on incorporation of radiolabeled precursors of DNA, RNA, protein, cell wall and lipid synthesis ([3H] thymidine, [3H] uridine, [3H] leucine, [14C] N-acetylglucosamine and [3H] glycerol, respectively) were quantified in *S. aureus* ATCC 29213. Results are expressed as percent of inhibition calculated based on the incorporation of each radiolabeled precursor. Statistical analyses were done using the two-tailed Student’s‘t’ test. *P* values of (*≤0.05) are considered as significant. Detailed “’P” values are listed below. DNA synthesis: control vs ciprofloxacin (8×):0.003, control vs simvastatin (0.25×):0.0023, control vs simvastatin (0.5×):0.0010, control vs simvastatin (1×):0.0003, control vs simvastatin (2×):0.0001, control vs simvastatin (4×):0.0008, control vs simvastatin (8×):0.0001. RNA synthesis: control vs rifampicin (8×):0.0006, control vs simvastatin (0.5×):0.0063, control vs simvastatin (1×):0.0005, control vs simvastatin (2×):0.0032, control vs simvastatin (4×):0.0003, control vs simvastatin (8×):0.0024. Protein synthesis: control vs linezolid (8×):0.0001, control vs simvastatin (0.25×):0.0098, control vs simvastatin (0.5×):0.0022, control vs simvastatin (1×):0.0006, control vs simvastatin (2×):0.0004, control vs simvastatin (4×):0.0004, control vs simvastatin (8×):0.0001. Cell wall synthesis: control vs vancomycin (8×):0.0001, control vs simvastatin (1×):0.0009, control vs simvastatin (2×):0.0005, control vs simvastatin (4×):0.0038, control vs simvastatin (8×):0.0004. Lipid synthesis: control vs cerulenin (8×):0.0001, control vs simvastatin (0.25×):0.0258, control vs simvastatin (0.5×):0.0040, control vs simvastatin (1×):0.0001, control vs simvastatin (2×):0.0001, control vs simvastatin (4×):0.0001, control vs simvastatin (8×):0.0004.

Simvastatin’s antibacterial mechanism of action was investigated using a standard macromolecular synthesis inhibition assay in *S. aureus* ATCC 29213. As shown in Fig. 1, DNA, protein and lipid synthesis were significantly inhibited at concentrations below the drug’s MIC (0.25×). In addition, simvastatin also significantly inhibited RNA synthesis at 0.5 × MIC. Inhibition of cell wall synthesis was observed only at the MIC.

### Simvastatin causes extensive protein degradation and disrupts cellular homeostasis

**Figure 2:**
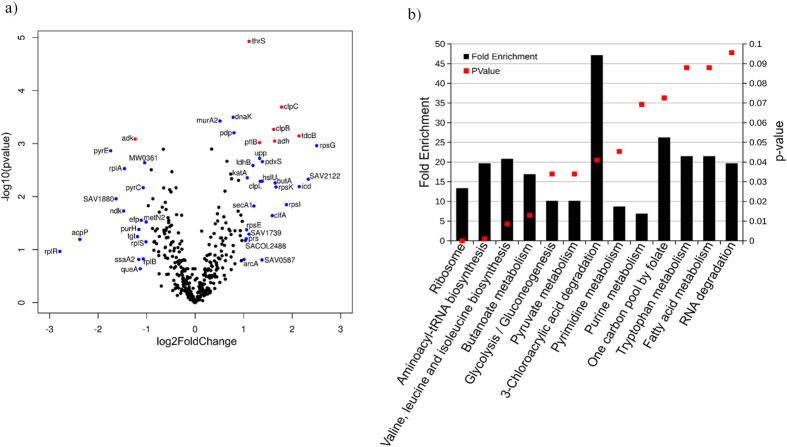
Quantitative proteome analysis of *S. aureus* cells treated with simvastatin reveals extensive protein degradation. (**a**) *S. aureus* treated with simvastatin in biological triplicates was analyzed for changes in the global proteome in relation to untreated controls, as shown in the volcano plot. The volcano plot depicts the *P*-values (−log_10_) versus gene ratio in the simvastatin-treated group (log_2_). Genes marked in blue indicate an absolute fold change higher than 1. The genes marked in red represent an adjusted *P*-value lower than 0.05 and an absolute fold change higher than 1.5. (**b**) Function–enrichment analysis of proteins degraded by simvastatin were annotated using Database for Annotation, Visualization and Integrated Discovery (DAVID). The overrepresented pathways are shown in columns and their *P*-values are represented by the red dots.

In order to gain additional insight into the different cellular pathways regulated by simvastatin, proteomic profiling was employed to investigate the response of bacteria to simvastatin^18^,^19^,^20^. The alterations in the proteome caused by treatment with simvastatin were compared to an untreated control group. The proteomic analysis identified 521 proteins with 85 proteins that were significantly differentially expressed (P ≤ 0.05) in the simvastatin treatment group as compared to the control group (Fig. 2a). The seven proteins marked in red have an adjusted P-value lower than 0.05 and absolute fold change higher than 1.5. An important protein that is regulated is adenylate kinase (adk) which is involved in the interconversion of ADP to AMP and ATP and helps to maintain the adenine nucleotide balance within cells^21^. From the six upregulated proteins, three are ATP-dependent enzymes; clpC (ATP-dependent Clp protease), clpB (chaperone protein ClpB) and thrS (threonine-tRNA ligase). The Clp proteases and chaperon proteins are central components in bacteria necessary to help mount an appropriate stress response to cope with adverse conditions experienced inside the host^22^,^23^.

The function-enrichment analysis found eight pathways showed a significant (P ≤ 0.05) fold enrichment ranging from 8.6 to 47 (Fig. 2b). From these pathways, the proteins involved in pyrimidine metabolism, valine, leucine and isoleucine biosynthesis and aminoacyl-tRNA biosynthesis were significantly downregulated (average log_2_ fold change: −1.42, −0.29 and −0.11 respectively). On the other hand, the proteome involved in 3-chloroacrylic acid degradation, butanoate metabolism, glycolysis/gluconeogenesis, pyruvate metabolism and the proteins that bind to one or more ribosomal subunits were significantly upregulated (average log_2_ fold change: 1.98, 1.26, 1.26, 0.82 and 0.61 respectively). Thus the proteomic analysis suggests that simvastatin treatment leads to an extensive degradation of different proteins involved in various essential cellular pathways resulting in dysregulation of cellular homeostasis and ultimately leading to arrest of bacterial growth.

### Simvastatin inhibits bacterial but not mammalian protein synthesis

**Figure 3:**
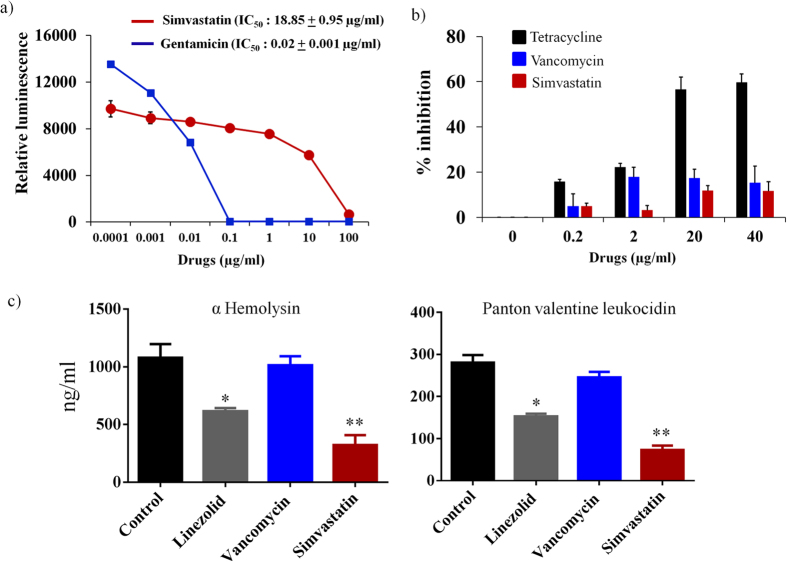
Simvastatin inhibits bacterial protein synthesis and toxin production. (**a**) Transcription-translation (TT) assay was carried out using S30 extracts from *E. coli*.IC_50_ of simvastatin and gentamicin required to inhibit 50% TT-activity in bacteria were determined. (**b**) Effect of simvastatin, vancomycin and tetracycline on mammalian mitobiogenesis was assessed via In cell- ELISA. J774A.1 cells were treated with indicated concentration of drugs and the levels of mitochondrial (mt)-DNA encoded protein (COX-I) and nuclear-DNA encoded protein (SDH-A) were quantified. The ratio of COX-I and SDH-A was calculated and the results shown are percent inhibition of mitochondrial biogenesis. (**c**) Effect of simvastatin on *S. aureus* toxin production. MRSA USA300 was treated with drugs for one hour and toxin production (ng/ml) (corrected for organism burden) was measured by ELISA. The results are given as means ± SD (n = 3). *P* values of (*P ≤ 0.05) (**P ≤ 0.01) are considered as significant in comparison to control groups. Detailed “P” values are listed below. α-hemolysin: control vs linezolid: 0.0318, control vs simvastatin: 0.0017. Panton-Valentine leukocidin: control vs linezolid: 0.0115, control vs simvastatin: 0.0052.

In order to confirm simvastatin is a potent, selective inhibitor of bacterial protein synthesis, its activity against both bacterial and mammalian mitochondrial protein synthesis was assessed. An *E. coli* S30 coupled transcription and translation assay was performed to determine the concentration of simvastatin required to inhibit 50% of the bacterial translational process (IC_50_). As presented in Fig. 3a, the IC_50_ of simvastatin was found to be 18.85 ± 0.95 μg/ml.

The effect of simvastatin on mammalian mitochondrial protein synthesis was subsequently evaluated in J774A.1 cells. The change in expression level of subunit I of Complex IV (COX-I), which is mitochondrial DNA (mtDNA)-encoded, and the 70 kDa subunit of Complex II (SDH-A), which is nuclear DNA (nDNA)-encoded proteins, after treatment with simvastatin and control antibiotics (tetracycline and vancomycin) was measured by In-cell ELISA. As presented in Fig. 3b, simvastatin (40 μg/ml), similar to vancomycin (40 μg/ml), has a very minimal effect (less than 15% inhibition observed) on inhibition of mitochondrial protein synthesis (Fig. 3b). In contrast, the positive control antibiotic, tetracycline, inhibited more than 50% of mitochondrial protein synthesis, at a concentration of 40 μg/ml (Fig. 3b).

### Simvastatin inhibits *S. aureus* toxin production

In view of results demonstrating the specific effect of simvastatin on bacterial protein synthesis inhibition, its effect on production of *S. aureus* toxins such as Panton-Valentine leucocidin (PVL) and α-hemolysin (Hla) was investigated using ELISA. Simvastatin significantly suppressed two key toxins (PVL and Hla) produced by MRSA USA300 when compared to the control group. This mimics the results obtained with linezolid (an antibiotic that inhibits protein synthesis) which also significantly suppressed production of both PVL and Hla by MRSA USA300 (Fig. 3c).

### Simvastatin effectively reduces pre-formed staphylococcal biofilms

**Figure 4:**
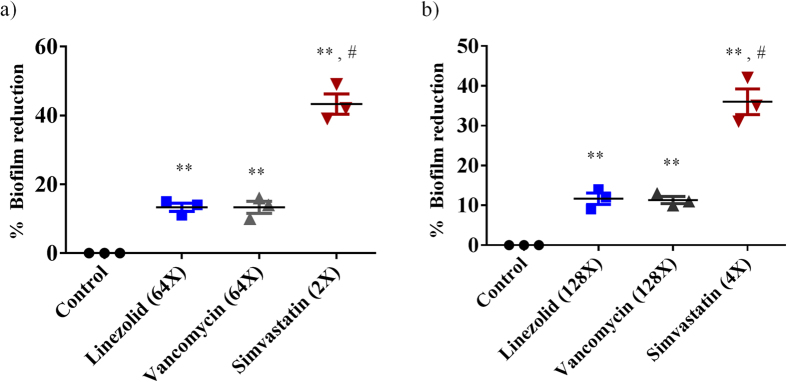
The effects of simvastatin and antibiotics (linezolid and vancomycin) on established biofilms of *S. aureus* (**a**) or *S. epidermidis* (**b**) were evaluated. The pre-formed biofilms were treated with control antibiotics or simvastatin and then stained with crystal violet. The optical density of the dissolved crystal violet was measured using a spectrophotometer. Values are the mean of triplicate samples with standard deviation bars. *P* values of (^*#^*P* ≤ 0.05) are considered as significant. (*) indicates simvastatin was compared to control and (#) to control antibiotics. Detailed *P* values are listed: *S. aureus* (**a**): Control vs linezolid, 0.0310; Control vs vancomycin, 0.0211; Control vs simvastatin: 0.0032; linezolid vs simvastatin, 0.0101; vancomycin vs simvastatin, 0.009. *S. epidermidis* (**b**): Control vs linezolid, 0.0220; Control vs vancomycin, 0.0171; Control vs simvastatin: 0.0021; linezolid vs simvastatin, 0.0110; vancomycin vs simvastatin, 0.0120.

Given the challenge associated with bacterial biofilms and their role in promoting recurring infection in hosts, we next moved to investigate the effect of simvastatin on disrupting established biofilms caused by *S. aureus* and *S. epidermidis*. Utilizing the microtiter dish biofilm formation assay, simvastatin was found to be capable of significantly reducing the adherent biofilms of both *S. aureus* and *S. epidermidis* when compared to conventional antibiotics (linezolid and vancomycin) (Fig. 4). Simvastatin, at 2 × MIC and 4 × MIC, significantly reduced *S. aureus* and *S. epidermidis* biofilm mass by approximately 40%. Contrary to simvastatin, the control antibiotics (linezolid and vancomycin) even at 64 × MIC and 128 × MIC were only able to reduce the biofilm mass of both *S. aureus* and *S. epidermidis* by 10%.

### Simvastatin is effective in reducing bacterial load in a mouse model of MRSA skin infection

**Figure 5:**
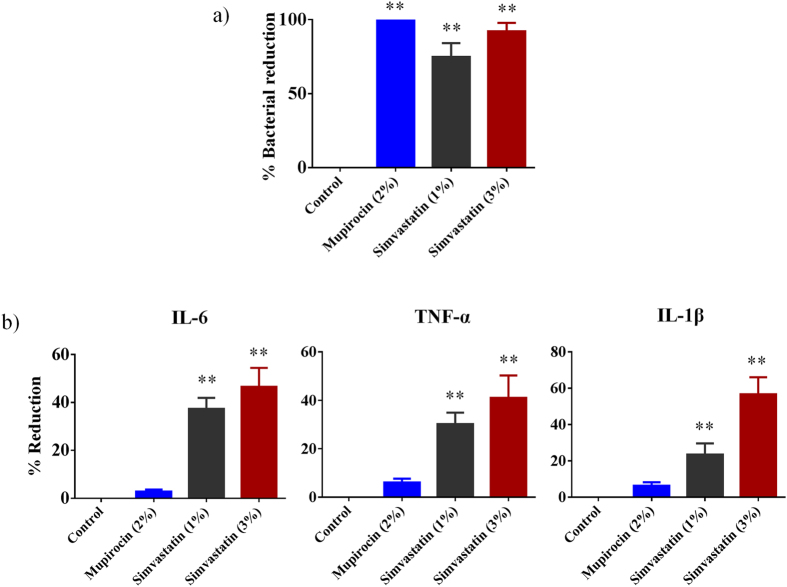
Antibacterial and anti-inflammatory activities of simvastatin in a mouse model of MRSA skin infection. (**a**) Efficacy of treatment of MRSA skin lesions with simvastatin (1 and 3%), mupirocin (2%) and petroleum jelly (negative control) once daily for four days. Percent bacterial reduction was calculated and shown in the figure. Statistical analysis was performed via the two-tailed Student *t* test. *P* values of (**P ≤ 0.01) are considered as significant. Detailed *P* values are listed: Control vs mupirocin (2%), <0.0001; Control vs simvastatin (1%), <0.0001; Control vs simvastatin (3%), <0.0001. (**b**) Effect of simvastatin on cytokines production in supernatants from skin homogenates of MRSA skin lesions. Percent reduction in inflammatory cytokines was calculated. Statistical analysis was performed via the two-tailed Student *t* test. *P* values of (**P ≤ 0.01) are considered as significant. Detailed *P* values are listed: IL-6: Control vs simvastatin (1%), 0.0013; Control vs simvastatin (3%), 0.0041. TNF-α: Control vs simvastatin (1%), 0.0030; Control vs simvastatin (3%), 0.0166. IL-1β: Control vs simvastatin (1%), 0.0205; Control vs simvastatin (3%), 0.0037.

Four groups of MRSA-infected mice were treated topically either with simvastatin (1% or 3%), a control antibiotic (2% mupirocin), or the vehicle alone (petroleum jelly) once a day for four days. As shown in Fig. 5a, all treatment groups significantly reduced the mean bacterial counts compared with the control group (*P* ≤ 0.01). Topical treatment with 1 and 3% simvastatin significantly reduced the MRSA load in infected skin wounds by 75 and 90% respectively. Mupirocin (2%) produced a 99% reduction in mean bacterial count as compared to the untreated group.

### Simvastatin reduces inflammatory cytokines induced by MRSA skin infection

The immune-modulatory activity of simvastatin against MRSA skin infection was evaluated by measuring levels of pro-inflammatory cytokines produced during infection including tumor necrosis factor-α (TNF-α), interleukin-6 (IL-6) and interleukin-1 beta (IL-1β) in the MRSA infected wounds of mice from the skin infection experiment described above. As shown in Fig. 5b, topical application of simvastatin (1 and 3%) significantly reduced all tested inflammatory cytokines. Simvastatin-treated (3%) group reduced production of all three cytokines examined (IL-6, TNF-α and IL-1β). Topical application of 1% simvastatin also decreased production of inflammatory cytokines in the MRSA infected wound lesions by 20%. However, mice treated with mupirocin (2%) did not show a significant reduction in the levels of all the tested inflammatory cytokines when compared to the control group.

### Simvastatin exhibits synergistic activity with conventional topical antimicrobials

**Figure 6:**
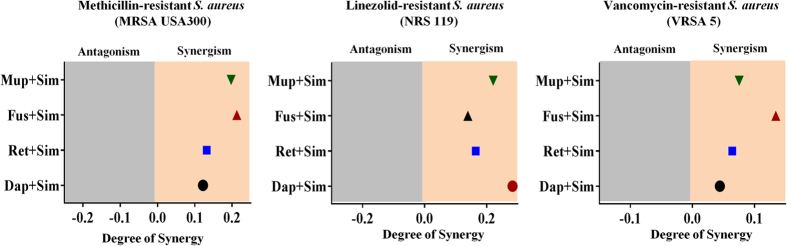
Synergistic activity of simvastatin with topical antimicrobials. The Bliss independence model confirms a synergistic relationship between simvastatin and four topical antimicrobials (mupirocin, fusidic acid, retapamulin and daptomycin) against various clinical isolates of multidrug-resistant strains of *S. aureus*. The positive and negative values along the x-axis represent the degree of synergism and antagonism respectively.

Combination therapy employing two or more antibiotics together has been utilized for treating skin wounds and infections in the healthcare setting. Given simvastatin exhibited good antibacterial activity against MRSA both *in vitro* and *in vivo*, we examined the possibility of using simvastatin with antimicrobials commonly used to treat skin infections. The antimicrobial activity of simvastatin in combination with four topical antimicrobials (fusidic acid, mupirocin, daptomycin, and retapamulin) was investigated *in vitro* using the Bliss independence model of synergism against three *S. aureus* clinical isolates. As shown in Fig. 6, simvastatin demonstrated a synergistic relationship with all tested topical antibiotics against *S. aureus* clinical isolates.

## Discussion

Antibiotics have long been key allies in the treatment of bacterial infections. However, the emergence of pathogens (in particular MRSA) exhibiting resistance to many antimicrobial classes including to therapeutic agents of last resort, such as vancomycin and linezolid, presents an ominous premonition that our current arsenal of antibiotics will no longer be effective in the near future^24^,^25^,^26^. Thus there is an urgent need to drive research efforts to discover new antimicrobials in order to circumvent this burgeoning health challenge. The conventional strategies used to develop new drugs are highly unlikely to keep pace with acquired resistance by bacterial pathogens and often comes at a significant financial risk to pharmaceutical companies (the success rate of receiving regulatory approval for a new antibiotic varies between 1.5–3.5% even after investing nearly one billion dollars in research and development costs). Though government regulatory agencies have attempted to provide incentives to encourage pharmaceutical companies to re-enter the arena of antibacterial drug discovery, such as the U.S. Food and Drug Administration’s “reboot” pledge, it will take many years for these incentives to translate into the discovery of new antibiotics (using conventional methods of screening compound libraries for lead hits)^27^. An alternative strategy that has promise to expedite the discovery and approval process is repurposing old drugs, such as statins that have already passed rigorous safety assessments, as novel antibacterial agents to combat multidrug-resistant pathogens.

Statins, widely used to control hyperlipidemia, are known to exhibit antimicrobial properties^9^,^10^,^11^. We investigated the antibacterial activity of eight statin drugs including simvastatin, atorvastatin, fluvastatin, lovastatin, mevastatin, pitavastatin, pravastatin and rosuvastatin against a representative Gram-positive and Gram-negative bacterial species (methicillin-resistant *S. aureus* ATCC 43300 and *P. aeruginosa* ATCC 15442). Our results correlate with previous reports that have found that only simvastatin exhibits antibacterial activity against Gram-positive bacteria^11^. However its activity against Gram-negative bacteria was previously unknown. Our initial investigation indicated that simvastatin lacks antibacterial activity against the Gram-negative pathogen *P. aeruginosa*. However, further analysis revealed that the outer membrane in Gram-negative bacteria acts as an intrinsic barrier for simvastatin to gain entry into Gram-negative bacteria. When the OM is compromised using a sub-inhibitory concentration of colistin, simvastatin exhibits antibacterial activity against many clinically-pertinent Gram-negative pathogens including *A. baumannii*, *E. coli, S. Typhimurium, K. pneumoniae, and P. aeruginosa*. The enhanced antimicrobial activity of simvastatin in comparison to other statin drugs may be related to differences in their chemical characteristics, as described previously^9^,^11^. However, further structure-activity relationship studies need to be performed to confirm the structural elements in simvastatin that contribute to its antimicrobial properties. This will permit rational modifications to be made to the drug’s structure in order to potentially enhance its potency against bacterial pathogens and mitigate potential toxicity issues to host tissues.

In view of the broad-spectrum activity of simvastatin, its antibacterial mode of action was investigated. Simvastatin exerts its antihyperlipidemic effect in humans by inhibiting the enzyme class I HMG-CoA reductase present in the mevalonate pathway^2^,^3^. We hypothesized that the mechanism of action (MOA) of simvastatin in *S. aureus* differs from the MOA in humans due to the absence of the class I HMG-CoA reductase enzyme in *S. aureus*^28^. In order to confirm this hypothesis, we tested the activity of simvastatin on *S. aureus* cultures supplemented with mevalonate. As expected, mevalonate supplementation (0.1 and 1 mM) did not diminish simvastatin’s antibacterial activity against *S. aureus* (data not shown). This clearly indicates that the MOA of simvastatin differs between *S. aureus* and humans. In order to further explore the MOA of simvastatin on *S. aureus*, a macromolecular synthesis assay was performed. Treatment of *S. aureus* cells with a subinhibitory concentration of simvastatin resulted in the suppression of multiple biosynthetic pathways including DNA, protein, lipid and RNA synthesis indicating that simvastatin might have a complex mechanism of action involving multiple targets. Additionally, the impact of simvastatin on multiple biosynthetic pathways might be due to dysregulation in pathways involved in general cellular homeostasis and energy metabolism such as glycolysis, pyruvate metabolism and butanoate metabolism as observed in the proteomic profiling. In order to ascertain whether cell membrane damage is the cause for inhibition of multiple macromolecular synthesis pathways, as noticed in antimicrobial peptides such as lactoferricin B and pleurocidin-derived peptides^29^,^30^, we performed an ATP release assay. Our results strongly suggest that simvastatin does not physically damage the bacterial cell membrane as was validated using transmission electron microscopy (see Supplementary Fig. S1 online). Future studies are needed to elucidate the exact molecular target(s) of simvastatin by which it exerts its antibacterial activity.

The macromolecular synthesis assay revealed that simvastatin inhibits bacterial protein synthesis which raises an important question; is this action specific or can simvastatin also inhibit protein synthesis in mammalian cells? Multiple antibacterials that inhibit bacterial protein synthesis (including tetracycline, linezolid and chloramphenicol) are non-selective and result in toxicity to the mitochondria in mammalian cells (given the similarity between the ribosomal subunits involved in protein synthesis in bacterial and human cells)^31^,^32^. When simvastatin’s ability to inhibit protein synthesis was further examined it was found that, unlike tetracycline which had a profound impact on inhibiting mitochondrial protein synthesis, simvastatin was a selective inhibitor of bacterial protein synthesis. The discovery led us to examine if this effect on protein synthesis inhibition would lead to suppression in the production of key toxins by *S. aureus*. Utilizing ELISA, we found that simvastatin is capable of inhibiting production of both PVL and αHla, two pore-forming cytotoxins that injure host immune cells and promote infection of host tissues^33^.

Confirmation of simvastatin’s broad spectrum antimicrobial activity *in vitro* led us to proceed forward with an *in vivo* experiment in a mouse model of MRSA infection. However, given simvastatin’s high MIC value cannot be achieved systemically, this limits the application of this drug to being used as a topical agent^34^. Due to the fact that *S. aureus* causes the vast majority of skin infections in humans and there is a demand for topical antimicrobial agents to treat these infections (given increasing resistance to first-line agents such as mupirocin), there is great potential for using simvastatin to treat/prevent bacterial infections in wounds^35^,^36^. Therefore we assessed the effectiveness of simvastatin as a topical antibacterial in a MRSA skin infection mouse model. Simvastatin, both at 1% and 3%, significantly reduced the mean MRSA counts compared with the control group (*P* ≤ 0.01), producing a 90% reduction in bacterial burden at the higher concentration. Thus, this skin infection study appears to strongly suggest that simvastatin has potential use as a topical antimicrobial for treatment of MRSA skin infections.

The clinical severity of *S. aureus*-based skin infections is driven in large part by production of excess host pro-inflammatory cytokines more so than by bacterial burden^37^,^38^. As simvastatin has known anti-inflammatory properties, it should be superior to traditional antibiotics for treatment of skin infection (as it should hypothetically suppress production of inflammatory cytokines)^39^. To confirm this, we measured the levels of three inflammatory cytokines in the supernatant of homogenized skin tissues obtained from the MRSA murine skin infection experiment described above. As predicted, topical treatment with simvastatin, both at 1 and 3%, significantly reduced production of three inflammatory cytokines (IL-1β, IL-6 and TNF-α); the suppression of these cytokines may contribute to enhanced healing of infected wounds^40^,^41^. Prolonged inflammation, especially due to the presence of inflammatory cytokines such as TNF-α and IL-6, delays healing in chronic infected wounds^42^. Simvastatin significantly (*P* ≤ 0.01) inhibits both cytokines (TNF-α and IL-6), which should provide a favorable outcome in wound healing^42^. Additionally, simvastatin has been shown to play a beneficial role in the healing process of diabetic and infected wounds by enhancing the formation of new blood and lymphatic vessels and increasing the formation of new tissue; these three effects undoubtedly confer an added advantage for using simvastatin to treat bacterial skin infections^43^,^44^.

Recurring infection in skin wounds can persist and impair wound healing due to the presence of complex microbial communities called biofilms. Bacterial biofilms, contribute significantly to the treatment failure of staph infections, due to hindering penetration of antibacterial drugs^45^. Simvastatin has been previously reported to exhibit anti-biofilm activity as it inhibited both growing and mature biofilms of *Candida* spp. and *Cryptococcus* spp^46^,^47^. Thus we decided to examine simvastatin’s capability to disrupt staphylococcal biofilms given their prevalence in the healthcare setting (in particular on medical implant devices). In addition to its broad-spectrum antibacterial activity, we confirmed that simvastatin is capable of disrupting established bacterial biofilms of two leading cause of hospital-acquired implant-based infections caused (*S. aureus* and *S. epidermidis*)^11^. The ability to disrupt staphylococcal biofilms by simvastatin lends further support to its potential use as a topical agent in the treatment of skin wounds.

The final component of the present study involved examining simvastatin’s ability to be used in combination with other topical antimicrobials. Due to the increasing incidence of MRSA strains demonstrating resistance to topical drugs of choice, such as fusidic acid and mupirocin, combination therapies are being explored as a potential mechanism to ward off the emergence of further resistance to these important agents^48^. The Bliss independence model was utilized to investigate if simvastatin has the potential to act synergistically with topical drug of choice against multidrug-resistant *S. aureus*^49^. Simvastatin behaved synergistically with fusidic acid, mupirocin, daptomycin, and retapamulin against *S. aureus* strains resistant to vancomycin, linezolid, and methicillin. This result provides a strong platform to further examine combining simvastatin with topical antimicrobials to treat staphylococcal skin infections (and potentially contribute to reducing the likelihood of strains developing resistance to each agent if used alone).

In conclusion, the present study builds upon previous reports that demonstrate simvastatin possesses antimicrobial activity against important Gram-positive pathogens, in particular methicillin-resistant *S. aureus*. We confirmed that simvastatin does possess antibacterial activity against Gram-negative pathogens as well, once the barrier imposed by the outer membrane is permeabilized, a finding not previously known. The antibacterial mechanism of action of simvastatin appears to be complex and involve inhibition of multiple biosynthetic pathways and cellular processes, including selective interference with bacterial protein synthesis. This property appears to play an important role in simvastatin’s ability to suppress production of key toxins (α-hemolysin and PVL) critical to permit skin wounds infected by *S. aureus* to fully heal. A murine MRSA skin infection experiment revealed simvastatin is capable of significantly reducing the bacterial burden present in infected wounds. Additionally, simvastatin demonstrates the ability to disrupt adherent staphylococcal biofilms and to be used in combination with other topical antimicrobials currently employed to treat MRSA skin infections. Collectively the present study lays the foundation for further investigation of repurposing simvastatin as a topical antibacterial agent to treat skin infections caused by pathogens including MRSA.

## Materials and Methods

### Bacterial strains and reagents

Bacterial strains used in this study are presented in Tables 1 and 2. Mueller-Hinton broth (MHB), gentamicin and tetracycline were purchased from Sigma-Aldrich while mupirocin (Applichem), linezolid (Selleck Chemicals), and vancomycin hydrochloride (Gold Biotechnology) were acquired from other commercial vendors. Mannitol salt agar (MSA), Trypticase soy agar (TSA) and Trypticase soy broth (TSB) were purchased from Becton, Dickinson and Company (Cockeysville, MD). All statin drugs used in this study were purchased from Sigma-Aldrich (St. Louis, MO, USA), with the exception of pitavastatin and rosuvastatin which were obtained from Selleckchem (Houston, TX, USA).

### Antibacterial assays

The antibacterial activity (MIC) of all test agents was examined using the broth microdilution method as per the guidelines outlined by the Clinical and Laboratory Standards Institute (CLSI)^50^.

### Gram-negative bacteria outer membrane permeabilization assay

The MIC of simvastatin and control antibiotics, in the presence of a sub-inhibitory concentration of colistin, against Gram-negative bacteria was evaluated as described in the antibacterial assay section above.

### Macromolecular synthesis assay

The macromolecular synthesis assay was conducted as described elsewhere^51^. Briefly, *S. aureus* strain ATCC 29213 was grown in TSB, until it reached exponential phase (OD_600_ = 0.2 to 0.3), and then treated with different concentrations of simvastatin and control antibiotics (ciprofloxacin, rifampicin, linezolid, vancomycin and cerulenin). Bacterial cells treated with drugs were incubated at 37 °C for 30 min and the radio labeled precursors for DNA ([3H] thymidine (0.5 μCi)), RNA ([3H] uridine (0.5 μCi)), protein ([3H] leucine (1.0 μCi)), cell wall ([14C] N-acetylglucosamine (0.4 μCi)) and lipid synthesis ([3H] glycerol (0.5 μCi)) were added for each reaction. The incorporation of radiolabeled precursors was quantified and the results expressed as percent inhibition of each specific pathway examined.

### Proteomics assay

An overnight culture of MRSA USA300 was treated with 10 × MIC of simvastatin for one hour at 37 °C. Bacterial cells were centrifuged and sequence grade Lys-C/Trypsin (Promega) was used to enzymatically digest samples. Samples were reduced and alkylated prior to digestion. All trypsin digestions were carried out in a Barocycler NEP2320 (PBI) at 50 °C under 20 kpsi for two hours. After digestion, samples were cleaned using MicroSpin C18 columns (Nest Group, Inc.) and the resulting pellets were re-suspended in 97% H_2_O/3% ACN/0.1% FA. A small aliquot (5 μL) of sample was analyzed via nanoLC-MS/MS.

The WIFF files from MS analysis were processed using the MaxQuant computational proteomics platform version 1.5.2.8 (Cox and Mann, 2008). The peak list generated was screened against the *Staphylococcus aureus* (10972 entries reviewed) and *Bos taurus* (41521 entries unreviewed) sequence from UNIPROT retrieved on 04/10/2015, in addition to a common contaminants database. The following settings were used for MaxQuant: initial precursor and fragment mass tolerance set to 0.07 and 0.02 Da respectively, Minimum peptides length of seven amino-acid, data were analyzed with ‘Label-free quantification’ (LFQ) checked and the ‘Match between runs’ interval set to one min, the fasta databases were randomized and the protein FDR was set to 1%, enzyme trypsin allowing for two missed cleavages and three modifications per peptide, fixed modifications were carbamidomethyl (C), variable modifications were set to Acetyl (Protein N-term) and Oxidation (M).

The MaxQuant results were used in in-house script, and the average LFQ intensity values for the technical replicates were used for each sample. All the *Bos taurus* and the common contaminant proteins were removed. All the values were transformed [log_2_(x)] and the missing values were inputted using the average values of all samples. The volcano plot and statistical analyses were performed in the R environment (www.cran.r-project.org). A t-test was performed on the LFQ intensity and only proteins with *P* ≤ 0.05 were used for further analyses. A function-enrichment analysis of proteins was annotated using the Database for Annotation, Visualization and Integrated Discovery—DAVID^52^.

### Cell-free bacterial transcription/translation assay

The cell-free bacterial transcription/ translation assay was performed using *Escherichia coli* S30 System (Promega). The assay was carried out as per the manufacturer’s instructions. Gentamicin was used as a positive control. Briefly, simvastatin and gentamicin were added at the indicated concentrations to the reaction mixtures and incubated at 37 °C for one hour. The intensity of luminescence was quantified using a standard FLx800 microplate reader (BioTek Instruments, Inc. Winooski, Vermont) after addition of the luciferase assay reagent.

### Mitochondrial biogenesis assay

An In-Cell ELISA Kit (MitoSciences Inc., Eugene, OR) was employed to evaluate the effect of simvastatin and control antibiotics (tetracycline and vancomycin) on mitochondrial protein synthesis and the experiment was conducted as described previously^53^. The ratio between COX-I and SDH-A was calculated and the percent inhibition of mitochondrial protein synthesis was determined.

### Measuring toxin production by ELISA

The effect of simvastatin and control antibiotics (linezolid and vancomycin) on production of two important *S. aureus* toxins (Hla and PVL) was measured utilizing ELISA as described elsewhere^51^,^54^,^55^.

### Mice infection

The animal care and all experiments were performed in accordance with the guidelines approved by Purdue University Animal Care and Use Committee (PACUC). The murine model of MRSA skin infection utilized in this study has been described previously^14^,^51^,^56^. Briefly, mice (eight week old female BALB/c mice, five mice per group) were injected intradermally with MRSA USA300 (1.65 × 108 CFU per mouse) and left for 48 h before an open wound formed at the injection site. Each group was subsequently treated with either 1% or 3% simvastatin or 2% mupirocin (using 20 mg petroleum jelly as the vehicle) once a day for four days. Control group was treated with the vehicle alone. 24 h after the last treatment, the area around the wound was lightly swabbed with 70% ethanol and the wound (1 cm^2^) was excised, homogenized, serially diluted, and plated on MSA. Plates were incubated at 37 °C for 18 hours before counting viable bacterial CFU.

### Quantifying inflammatory cytokines by ELISA

Skin homogenates obtained from the mice skin infection procedure described above were centrifuged and the supernatants were assayed in order to measure the levels of three cytokines TNF-α, IL-6 and IL-1β by Duo-set ELISA Kits (R&D Systems, Inc.) The quantification of cytokines and the experiment were carried out as per the manufacturer’s instructions^51^.

### Biofilm assay

The effect of simvastatin and control antibiotics (vancomycin and linezolid) on disrupting established staphylococcal biofilm was evaluated using the microtiter dish biofilm formation assay^51^,^57^. Briefly, *S. aureus* (ATCC 6538) and *S. epidermidis* (ATCC 35984) were grown in TSB supplemented with 1% glucose in a 96-well tissue-culture treated plate. Bacteria were incubated at 37 °C for 24 h to permit the formation of an adherent biofilm. The medium was removed and washed with PBS. Drugs at indicated concentration were added and incubated again at 37 °C for 24 h. Plates were washed again and biofilms were stained with 0.1% (wt/vol) crystal violet. Plates were washed, air dried and biofilm mass was dissolved using 95% ethanol. The intensity of crystal violet was measured using a micro plate reader (Bio-Tek Instruments Inc.). Data are presented as the percent biofilm mass reduction in treated groups in relation to untreated wells.

### Synergistic assay

Synergism was calculated using the Bliss independence model as described in previous reports^51^. Briefly, bacterial strains were incubated with a sub-inhibitory concentration of simvastatin and control antimicrobials for 12 h and the degree of synergy was calculated using the formula: *S = *(*f*_*A0*_*/f*_*00*_)(*f*_*0B*_*/f*_*00*_)−(*f*_*AB*_*/f*_*00*_), where *f*_*AB*_ refers to bacterial growth rate in the presence of the combined drugs at concentration *A*, for one of the antibiotics, and *B* for the simvastatin; *f*_*A0*_ and *f*_*0B*_ refer to the bacterial growth rates in the presence of antibiotics (or) simvastatin at a concentration of *A* and *B*, respectively; *f*_*00*_ refers to the bacterial growth rate in the absence of drugs. Positive values correlate with synergistic behavior while negative values are indicative of an antagonistic interaction between the drugs.

### Statistical analyses

Statistical analyses were assessed using GraphPad Prism 6.0 (Graph Pad Software, La Jolla, CA). *P* values were calculated using the two-tailed Student *t* test. P < 0.05 was deemed significant.

## Additional Information

**How to cite this article**: Thangamani, S. *et al*.Exploring simvastatin, an antihyperlipidemic drug, as a potential topical antibacterial agent. *Sci. Rep*.**5**, 16407; doi: 10.1038/srep16407 (2015).

## Supplementary Material

Supplementary Information

Supplementary Data

## Figures and Tables

**Table 1:** MIC of simvastatin against a panel of Gram-positive bacteria.

Bacteria (no. of strains screened)	Simvastatin (μg/ml)	
	MIC_50_	MIC_90_
Methicillin-resistant *S. aureus* (18)	32	32
Vancomycin-resistant *S. aureus* (15)	32	32
Methicillin-sensitive *S. aureus* (6)	32	32
Vancomycin-intermediate *S. aureus* (3)	32	32
Vancomycin-sensitive *Enterococcus* (9)	32	32
Vancomycin-resistant *Enterococcus* (7)	32	32
*Listeria monocytogenes* (6)	32	32
*Streptococcus pneumoniae* (2)	64	64
*Bacillus anthracis* (3)	16	16

**Table 2:** MIC of simvastatin against a panel of Gram-negative bacteria.

Bacterial strain	MIC ofcolistin(μg/ml)	Sub-inhibitory concentrationof colistin used(μg/ml)	Simvastatin(μg/ml)		Erythromycin(μg/ml)		Fusidic acid(μg/ml)		Daptomycin(μg/ml)	
			colistin		colistin		colistin		colistin	
			(−)	(+)	(−)	(+)	(−)	(+)	(−)	(+)
*Acinetobacter baumannii*ATCC BAA19606	0.25	0.0625	>256	16	64	2	64	0.5	>256	>256
*Acinetobacter baumannii*ATCC BAA1605	0.25	0.0625	>256	16	64	2	128	1	>256	>256
*Acinetobacter baumannii*ATCC BAA747	0.25	0.0625	>256	16	64	2	128	1	>256	>256
*Escherichia coli* O157:H7ATCC 700728	0.25	0.0625	>256	16	128	1	>256	4	>256	>256
*Escherichia coli* O157:H7ATCC 35150	0.125	0.0625	>256	8	128	4	>256	4	>256	>256
*Salmonella Tphimurium*ATCC 700720	1	0.25	>256	16	256	0.5	>256	0.5	>256	>256
*Klebsiella pneumoniae*ATCC BAA 2146	0.25	0.125	>256	16	>256	0.125	>256	0.125	>256	>256
*Klebsiella pneumoniae*ATCC BAA 1705	0.25	0.125	>256	16	>256	8	>256	8	>256	>256
*Pseudomonas aeruginosa*ATCC 9721	0.5	0.25	>256	16	>256	2	>256	1	>256	>256
*Pseudomonas aeruginosa*ATCC 9027	0.5	0.25	>256	32	>256	0.5	>256	2	>256	>256
*Pseudomonas aeruginosa*ATCC 27853	0.5	0.25	>256	16	256	0.5	>256	0.5	>256	>256
*Pseudomonas aeruginosa*ATCC BAA-1744	0.25	0.125	>256	16	>256	1	>256	2	>256	>256
*Pseudomonas aeruginosa*ATCC 25619	0.125	0.0625	>256	16	256	2	>256	0.5	>256	>256
*Pseudomonas aeruginosa*ATCC 35032	0.5	0.25	>256	16	>256	1	>256	1	>256	>256
*Pseudomonas aeruginosa*ATCC 10145	0.25	0.125	>256	16	256	1	>256	2	>256	>256
*Pseudomonas aeruginosa*ATCC 15442	0.5	0.25	>256	16	>256	0.5	>256	1	>256	>256
*Escherichia coli* 1411	0.25	0.0625	>256	16	32	0.03	>256	0.03	>256	>256
*Escherichia coli* SM1411∆ *acrAB*	0.25	0.0625	>256	16	0.03	<0.03	8	<0.03	>256	>256

## References

[b1] Cziraky M. J., Watson K. E. & Talbert R. L. Targeting low HDL-cholesterol to decrease residual cardiovascular risk in the managed care setting. J Manag Care Pharm 14, S3–28, quiz S30-21 (2008). 19891279 10.18553/jmcp.2008.14.S8-A.1 PMC10438150

[b2] Shitara Y. & Sugiyama Y. Pharmacokinetic and pharmacodynamic alterations of 3-hydroxy-3-methylglutaryl coenzyme A (HMG-CoA) reductase inhibitors: drug-drug interactions and interindividual differences in transporter and metabolic enzyme functions. Pharmacology & therapeutics 112, 71–105 (2006). 16714062 10.1016/j.pharmthera.2006.03.003

[b3] McTaggart F. .Preclinical and clinical pharmacology of Rosuvastatin, a new 3-hydroxy-3-methylglutaryl coenzyme A reductase inhibitor. The American journal of cardiology 87, 28B–32B (2001). 11256847 10.1016/s0002-9149(01)01454-0

[b4] Liao J. K. & Laufs U. Pleiotropic effects of statins. Annual review of pharmacology and toxicology 45, 89–118 (2005). 10.1146/annurev.pharmtox.45.120403.095748 PMC2694580 15822172

[b5] Parihar S. P. .Simvastatin enhances protection against Listeria monocytogenes infection in mice by counteracting Listeria-induced phagosomal escape. PloS one 8, e75490 (2013). 24086542 10.1371/journal.pone.0075490 PMC3782446

[b6] Parihar S. P. .Statin therapy reduces the mycobacterium tuberculosis burden in human macrophages and in mice by enhancing autophagy and phagosome maturation. The Journal of infectious diseases 209, 754–763 (2014). 24133190 10.1093/infdis/jit550

[b7] Janda S., Young A., Fitzgerald J. M., Etminan M. & Swiston J. The effect of statins on mortality from severe infections and sepsis: a systematic review and meta-analysis. Journal of critical care 25, 656 e657–622 (2010). 20413251 10.1016/j.jcrc.2010.02.013

[b8] Bjorkhem-Bergman L., Bergman P., Andersson J. & Lindh J. D. Statin treatment and mortality in bacterial infections-a systematic review and meta-analysis. PloS one 5, e10702 (2010). 20502712 10.1371/journal.pone.0010702 PMC2873291

[b9] Jerwood S. & Cohen J. Unexpected antimicrobial effect of statins. The Journal of antimicrobial chemotherapy 61, 362–364 (2008). 18086693 10.1093/jac/dkm496

[b10] Bergman P. .Studies on the antibacterial effects of statins-*in vitro* and *in vivo*. PloS one 6, e24394 (2011). 21912631 10.1371/journal.pone.0024394 PMC3166163

[b11] Graziano T. S. .Statins and Antimicrobial Effects: Simvastatin as a Potential Drug against Staphylococcus aureus Biofilm. PloS one 10, e0128098 (2015). 26020797 10.1371/journal.pone.0128098 PMC4447369

[b12] Lobato L. S. .Statins increase rifampin mycobactericidal effect. Antimicrobial agents and chemotherapy 58, 5766–5774 (2014). 25049257 10.1128/AAC.01826-13 PMC4187984

[b13] Pendleton J. N., Gorman S. P. & Gilmore B. F. Clinical relevance of the ESKAPE pathogens. Expert review of anti-infective therapy 11, 297–308 (2013). 23458769 10.1586/eri.13.12

[b14] Mohammad H., Thangamani S. & Seleem M. N. Antimicrobial peptides and peptidomimetics-potent therapeutic allies for staphylococcal infections. Current pharmaceutical design 21, 2073–2088 (2015). 25760338 10.2174/1381612821666150310102702 PMC8686049

[b15] Thangamani S., Mohammad H., Younis W. & Seleem M. N. Drug repurposing for the treatment of staphylococcal infections. Current pharmaceutical design 21, 2089–2100 (2015). 25760334 10.2174/1381612821666150310104416 PMC8672279

[b16] Thangamani S., Younis W. & Seleem M. N. Repurposing Clinical Molecule Ebselen to Combat Drug Resistant Pathogens. PloS one 10, e0133877 (2015). 26222252 10.1371/journal.pone.0133877 PMC4519285

[b17] Younis W., Thangamani S. & Seleem M. N. Repurposing Non-Antimicrobial Drugs and Clinical Molecules to Treat Bacterial Infections. Current pharmaceutical design 21, 4106–4111 (2015). 25961308 10.2174/1381612821666150506154434 PMC4686870

[b18] Lok C. N. .Proteomic analysis of the mode of antibacterial action of silver nanoparticles. Journal of proteome research 5, 916–924 (2006). 16602699 10.1021/pr0504079

[b19] Bandow J. E., Brotz H., Leichert L. I., Labischinski H. & Hecker M. Proteomic approach to understanding antibiotic action. Antimicrobial agents and chemotherapy 47, 948–955 (2003). 12604526 10.1128/AAC.47.3.948-955.2003 PMC149304

[b20] Wenzel M. .Proteomic signature of fatty acid biosynthesis inhibition available for *in vivo* mechanism-of-action studies. Antimicrobial agents and chemotherapy 55, 2590–2596 (2011). 21383089 10.1128/AAC.00078-11 PMC3101464

[b21] Jacobs A. C. .Adenylate kinase release as a high-throughput-screening-compatible reporter of bacterial lysis for identification of antibacterial agents. Antimicrobial agents and chemotherapy 57, 26–36 (2013). 23027196 10.1128/AAC.01640-12 PMC3535927

[b22] Chatterjee I., Neumayer D. & Herrmann M. Senescence of staphylococci: using functional genomics to unravel the roles of ClpC ATPase during late stationary phase. International journal of medical microbiology : IJMM 300, 130–136 (2010). 19931487 10.1016/j.ijmm.2009.10.004

[b23] Frees D., Gerth U. & Ingmer H. Clp chaperones and proteases are central in stress survival, virulence and antibiotic resistance of Staphylococcus aureus. International journal of medical microbiology : IJMM 304, 142–149 (2014). 24457183 10.1016/j.ijmm.2013.11.009

[b24] Graber C. J. .Intermediate vancomycin susceptibility in a community-associated MRSA clone. Emerging infectious diseases 13, 491–493 (2007). 17552110 10.3201/eid1303.060960 PMC2725904

[b25] Rybak M. J. .Characterization of vancomycin-heteroresistant Staphylococcus aureus from the metropolitan area of Detroit, Michigan, over a 22-year period (1986 to 2007). Journal of clinical microbiology 46, 2950–2954 (2008). 18632899 10.1128/JCM.00582-08 PMC2546725

[b26] Locke J. B. .Elevated linezolid resistance in clinical cfr-positive Staphylococcus aureus isolates is associated with co-occurring mutations in ribosomal protein L3. Antimicrobial agents and chemotherapy 54, 5352–5355 (2010). 20837755 10.1128/AAC.00714-10 PMC2981277

[b27] Shlaes D. M., Sahm D., Opiela C. & Spellberg B. The FDA reboot of antibiotic development. Antimicrob Agents Chemother 57, 4605–4607 (2013). 23896479 10.1128/AAC.01277-13 PMC3811409

[b28] Wilding E. I. .Essentiality, expression, and characterization of the class II 3-hydroxy-3-methylglutaryl coenzyme A reductase of Staphylococcus aureus. Journal of bacteriology 182, 5147–5152 (2000). 10960099 10.1128/jb.182.18.5147-5152.2000 PMC94663

[b29] Patrzykat A., Friedrich C. L., Zhang L., Mendoza V. & Hancock R. E. Sublethal concentrations of pleurocidin-derived antimicrobial peptides inhibit macromolecular synthesis in Escherichia coli. Antimicrobial agents and chemotherapy 46, 605–614 (2002). 11850238 10.1128/AAC.46.03.605-614.2002 PMC127508

[b30] Ulvatne H., Samuelsen O., Haukland H. H., Kramer M. & Vorland L. H. Lactoferricin B inhibits bacterial macromolecular synthesis in Escherichia coli and Bacillus subtilis. FEMS microbiology letters 237, 377–384 (2004). 15321686 10.1016/j.femsle.2004.07.001

[b31] McKee E. E., Ferguson M., Bentley A. T. & Marks T. A. Inhibition of mammalian mitochondrial protein synthesis by oxazolidinones. Antimicrobial agents and chemotherapy 50, 2042–2049 (2006). 16723564 10.1128/AAC.01411-05 PMC1479116

[b32] Parashar S., Rao R., Tikare S. K. & Tikare S. S. Chloramphenicol induced reversible bone marrow suppression. A case report. Journal of postgraduate medicine 18, 90–92 (1972). 5037129

[b33] DuMont A. L. & Torres V. J. Cell targeting by the Staphylococcus aureus pore-forming toxins: it’s not just about lipids. Trends in microbiology 22, 21–27 (2014). 24231517 10.1016/j.tim.2013.10.004 PMC3929396

[b34] Lennernas H. & Fager G. Pharmacodynamics and pharmacokinetics of the HMG-CoA reductase inhibitors. Similarities and differences. Clinical pharmacokinetics 32, 403–425 (1997). 9160173 10.2165/00003088-199732050-00005

[b35] Otter J. A. & French G. L. Molecular epidemiology of community-associated meticillin-resistant Staphylococcus aureus in Europe. The Lancet. Infectious diseases 10, 227–239 (2010). 20334846 10.1016/S1473-3099(10)70053-0

[b36] Simor A. E. .Mupirocin-resistant, methicillin-resistant Staphylococcus aureus strains in Canadian hospitals. Antimicrobial agents and chemotherapy 51, 3880–3886 (2007). 17724154 10.1128/AAC.00846-07 PMC2151460

[b37] Montgomery C. P. .Local inflammation exacerbates the severity of Staphylococcus aureus skin infection. PloS one 8, e69508 (2013). 23861974 10.1371/journal.pone.0069508 PMC3702601

[b38] Sharma-Kuinkel B. K., Zhang Y., Yan Q., Ahn S. H. & Fowler V. G. Jr. Host gene expression profiling and *in vivo* cytokine studies to characterize the role of linezolid and vancomycin in methicillin-resistant Staphylococcus aureus (MRSA) murine sepsis model. PloS one 8, e60463 (2013). 23565251 10.1371/journal.pone.0060463 PMC3614971

[b39] Lefer D. J. Statins as potent antiinflammatory drugs. Circulation 106, 2041–2042 (2002). 12379569 10.1161/01.cir.0000033635.42612.88

[b40] Jialal I., Miguelino E., Griffen S. C. & Devaraj S. Concomitant reduction of low-density lipoprotein-cholesterol and biomarkers of inflammation with low-dose simvastatin therapy in patients with type 1 diabetes. The Journal of clinical endocrinology and metabolism 92, 3136–3140 (2007). 17519305 10.1210/jc.2007-0453 PMC2677961

[b41] Cowin A. J., Hatzirodos N., Rigden J., Fitridge R. & Belford D. A. Etanercept decreases tumor necrosis factor-alpha activity in chronic wound fluid. Wound repair and regeneration: official publication of the Wound Healing Society [and] the European Tissue Repair Society 14, 421–426 (2006). 10.1111/j.1743-6109.2006.00141.x 16939569

[b42] Fournier B. & Philpott D. J. Recognition of Staphylococcus aureus by the innate immune system. Clinical microbiology reviews 18, 521–540 (2005). 16020688 10.1128/CMR.18.3.521-540.2005 PMC1195972

[b43] Derici H. .Simvastatin Improves Incisional Wound Healing in a Rat Model: An Experimental Study. Wounds: a compendium of clinical research and practice 24, 195–200 (2012). 25874542

[b44] Farsaei S., Khalili H. & Farboud E. S. Potential role of statins on wound healing: review of the literature. International wound journal 9, 238–247 (2012). 22050652 10.1111/j.1742-481X.2011.00888.x PMC7950468

[b45] Mohamed M. F., Hamed M. I., Panitch A. & Seleem M. N. Targeting Methicillin-Resistant Staphylococcus aureus with Short Salt-Resistant Synthetic Peptides. Antimicrobial agents and chemotherapy 58, 4113–4122 (2014). 24798285 10.1128/AAC.02578-14 PMC4068565

[b46] Liu G., Vellucci V. F., Kyc S. & Hostetter M. K. Simvastatin inhibits Candida albicans biofilm *in vitro*. Pediatric research 66, 600–604 (2009). 19707174 10.1203/PDR.0b013e3181bd5bf8

[b47] Brilhante R. S. .Simvastatin inhibits planktonic cells and biofilms of Candida and Cryptococcus species. The Brazilian journal of infectious diseases: an official publication of the Brazilian Society of Infectious Diseases 19, 459–465 (2015). 10.1016/j.bjid.2015.06.001 PMC9427464 26119850

[b48] Huang L., Dai T., Xuan Y., Tegos G. P. & Hamblin M. R. Synergistic combination of chitosan acetate with nanoparticle silver as a topical antimicrobial: efficacy against bacterial burn infections. Antimicrobial agents and chemotherapy 55, 3432–3438 (2011). 21502618 10.1128/AAC.01803-10 PMC3122390

[b49] Morones-Ramirez J. R., Winkler J. A., Spina C. S. & Collins J. J. Silver enhances antibiotic activity against gram-negative bacteria. Science translational medicine 5, 190ra181 (2013). 10.1126/scitranslmed.3006276 PMC3771099 23785037

[b50] CLSI. Methods for dilution antimicrobial susceptibility tests for bacteria that grow aerobically; approved standard M7-A7. CLSI, Wayne, PA. (2007).

[b51] Thangamani S., Younis W. & Seleem M. N. Repurposing ebselen for treatment of multidrug-resistant staphylococcal infections. Scientific Reports 5, 11596 (2015). 26111644 10.1038/srep11596 PMC4481386

[b52] Huang da W., Sherman B. T. & Lempicki R. A. Bioinformatics enrichment tools: paths toward the comprehensive functional analysis of large gene lists. Nucleic acids research 37, 1–13 (2009). 19033363 10.1093/nar/gkn923 PMC2615629

[b53] Cohen D. J. .Postoperative intraperitoneal 5-fluoro-2′-deoxyuridine added to chemoradiation in patients curatively resected (R0) for locally advanced gastric and gastroesophageal junction adenocarcinoma. Annals of surgical oncology 19, 478–485 (2012). 21769462 10.1245/s10434-011-1940-8

[b54] Stevens D. L., Maier K. A. & Mitten J. E. Effect of antibiotics on toxin production and viability of Clostridium perfringens. Antimicrobial agents and chemotherapy 31, 213–218 (1987). 2882731 10.1128/aac.31.2.213 PMC174694

[b55] Stevens D. L. .Impact of antibiotics on expression of virulence-associated exotoxin genes in methicillin-sensitive and methicillin-resistant Staphylococcus aureus. The Journal of infectious diseases 195, 202–211 (2007). 17191165 10.1086/510396

[b56] Mohamed M. F. & Seleem M. N. Efficacy of short novel antimicrobial and anti-inflammatory peptides in a mouse model of methicillin-resistant Staphylococcus aureus (MRSA) skin infection. Drug design, development and therapy 8, 1979–1983 (2014). 10.2147/DDDT.S72129 PMC4207544 25378910

[b57] Mohammad H., Mayhoub A. S., Cushman M. & Seleem M. N. Anti-biofilm activity and synergism of novel thiazole compounds with glycopeptide antibiotics against multidrug-resistant Staphylococci. The Journal of antibiotics 68, 259–266 (2015). 25315757 10.1038/ja.2014.142 PMC4429288

