# Supplementary material for: Exploring simvastatin, an antihyperlipidemic drug, as a potential topical antibacterial agent
Source: Sci Rep. 2015 Nov 10;5:16407. doi: 10.1038/srep16407 (PMC4639749; doi:10.1038/srep16407)

Supplementary materials

Exploring simvastatin, an antihyperlipidemic drug, as a potential topical antibacterial agent

Shankar Thangamani[1], Haroon Mohammad[1], Mostafa Abushahba[1], Maha Hamed[1], Tiago Sobreira[2], Victoria Hedrick[2], Lake Paul[2] and Mohamed Seleem[1]*

Supplementary methods:

ATP release assay: In order to determine if simvastatin and control antibiotics were capable of disrupting the MRSA cell membrane, MRSA USA300 cells were treated with 5 × MIC of simvastatin, tetracycline, or lysostaphin for one hour at 37°C. DMSO was used as a negative control. Bacteria were centrifuged and supernatants were analyzed using the Enliten ATP Assay System (Promega) per the manufacturer's instructions. Aliquots (10 µl) of supernatant were mixed with 75 µl of luciferase assay reagent and the intensity of luminescence was recorded using a microplate reader (FLx800 BioTek Instruments, Inc. Winooski, Vermont).

Electron Microscopy: An overnight culture of MRSA USA300 was diluted (OD_{600} = 0.3) and incubated with 5 × MIC of simvastatin before samples were subsequently collected at two time points (0 and 12 hours). Samples were centrifuged and the bacterial pellets were fixed with 2.5% buffered glutaraldehyde for one hour. Cells were next treated with 1% osmium tetroxide and 1% uranyl acetate. Further dehydration was done using ethanol and embedded in white resin. The samples were stained with 1% uranyl acetate and lead citrate prior to viewing samples under a Philips CM-100 microscope

Supplementary figure legends:

Supplementary figure S1. Simvastatin does not disrupt the cell membrane of *S. aureus*. (A) MRSA USA300 cells were treated with 5 × MIC of simvastatin, tetracycline or lysostaphin and the level of ATP was measured in the supernatant for each treatment condition. (B) Transmission electron microscopy (TEM) images of untreated and simvastatin (5 × MIC) treated MRSA USA300 cells at the indicated time points, in hours (h), are shown.

Supplementary Table S1: Screening statins for antibacterial activity

Statins/ Molecular formula	Pub Chem ID	M.wt	InChIKey	MRSA ATCC 4330 (µg/ml)	*P. aeruginosa* ATCC 15442 (µg/ml)
Simvastatin $C_{25}H_{38}O_5$	54454	418.56	RYMZZMVNJRMUD DHGQWONQESA-N	32	>1024
Atorvastatin $C_{33}H_{35}FN_2O_5$	60823	558.63	XUKUURHRXDUEB CKAYWLYCHSA-N	>1024	>1024
Fluvastatin $C_{24}H_{26}FNO_4$	446155	411.46	FJLGEFLZQAZZCD MCBHFWOFSA-N	>1024	>1024
Lovastatin $C_{24}H_{36}O_5$	53232	404.53	PCZOHLXUXFIOCF BXMDZJJMSA-N	>1024	>1024
Mevastatin $C_{23}H_{34}O_5$	64715	390.51	AJLFOPYRIVGYMJI NTXDZFKSA-N	>1024	>1024
Pitavastatin $C_{25}H_{24}FNO_4$	5282452	421.46	VGYFMXBACGZSI LMCBHFWOFSA-N	>1024	>1024
Pravastatin $C_{23}H_{36}O_7$	54687	424.52	TUZYXOIXSAXUG OPZAWKZKUSA-N	>1024	>1024
Rosuvastatin $C_{44}H_{54}CaF_2N_6O_{12}S_2$	5282455	1001.1	LALFOYNTGMUKG GBGRFNVSISA-L	>1024	>1024

Supplementary Table S2: MIC of simvastatin against *Staphylococcus* spp.

Strain type	Strain ID	Phenotypic properties	MIC/MBC (µg/ml)
Methicillin-sensitive *S. aureus* (MSSA)	ATCC 6538	Quality control and biofilm-forming strain	32/ >128
	RN4220		32/ >128
	NRS72	Resistant to penicillin	32/ >128
	NRS77		32/ >128
	NRS846		32/ >128
	NRS860		32/ >128
Methicillin resistant *S. aureus* (MRSA)	USA100	Resistant to ciprofloxacin, clindamycin,	32/ >128
		erythromycin	
	USA200	Resistant to clindamycin, methicillin	32/ >128
		erythromycin, gentamicin,	
	USA300	Resistant to erythromycin, methicillin,	32/ >128
		tetracycline	
	USA400	Resistant to methicillin, tetracycline	32/ >128
	USA500	Resistant to ciprofloxacin, clindamycin,	32/ >128
		erythromycin, gentamicin,	
		methicillin, tetracycline, trimethoprim	
	USA700	Resistant to erythromycin, methicillin	32/ >128
	USA800	Resistant to methicillin	32/ >128
	USA1000	Resistant to erythromycin, methicillin	32/ >128
	USA1100	Resistant to methicillin	32/ >128
	NRS194	Resistant to methicillin	64/ >128
	NRS108	Resistant to gentamicin	32/ >128
	NRS119 (Lin^{r})	Resistant to linezolid	32/ >128
	ATCC 43300	Resistant to methicillin	32/ >128
	ATCC BAA-44	Multidrug-resistant strain	32/ >128
	NRS70	Resistant to erythromycin, clindamycin	32/ >128
		spectinomycin	
	NRS71	Resistant to tetracycline, methicillin	32/ >128
	NRS100	Resistant to tetracycline, methicillin	64/ >128
	NRS123	Resistant to tetracycline, methicillin	32/ >128
Vancomycin-intermediate *S. aureus* (VISA)	NRS1	Resistant to aminoglycosides and tetracycline; glycopeptide-intermediate *S. aureus*	32/ >128
	NRS19	Glycopeptide-intermediate *S. aureus*	32/ >128
	NRS37	Glycopeptide-intermediate *S. aureus*	32/ >128
Vancomycin-resistant *S. aureus* (VRSA)	VRS1	Resistant to vancomycin	32/ >128
	VRS2	Resistant to vancomycin, erythromycin, spectinomycin	32/ >128
	VRS3a	Resistant to vancomycin	32/ >128
	VRS3b	Resistant to vancomycin	32/ >128
	VRS4	Resistant to vancomycin, erythromycin, spectinomycin	32/ >128
	VRS5	Resistant to vancomycin	32/ >128
	VRS6	Resistant to vancomycin	32/ >128
	VRS7	Resistant to vancomycin, β-lactams	32/ >128
	VRS8	Resistant to vancomycin	32/ >128
	VRS9	Resistant to vancomycin	64/ >128
	VRS10	Resistant to vancomycin	32/ >128
	VRS11a	Resistant to vancomycin	32/ >128
	VRS11b	Resistant to vancomycin	32/ >128
	VRS12	Resistant to vancomycin	32/ >128
	VRS13	Resistant to vancomycin	32/ >128
S. epidermidis	NRS101	Prototype biofilm producer; resistant to methicillin, gentamicin	32/ >128

Supplementary Table S3: MIC of simvastatin against *Enterococcus*, *Listeria*, *Streptococcus* and *Bacillus* spp.

Strain ID	Phenotypic Characteristics	MIC/MBC (µg/ml)
E. faecalis ATCC49533	Resistant to streptomycin	32/ >128
E. faecalis ATCC7080	-	32/ >128
E. faecalis ATCC49532	Resistant to gentamicin	32/ >128
E. faecalis ATCC14506	-	32/ >128
E. faecalis ATCC 51229 (VRE)	Resistant to Vancomycin. Sensitive to Teicoplanin	32/ >128
E. faecalis SF24397	Resistant to erythromycin (ermB+) and gentamicin	32/ >128
E. faecalis SF24413 (VRE)	Resistant to erythromycin, gentamicin and vancomycin.	32/ >128
E. faecalis SF28073 (VRE)	Resistant to erythromycin, gentamicin and vancomycin	32/ >128
E. faecalis HH22	Resistant to penicillin, erythromycin, tetracycline and high levels of aminoglycosides	32/ >128
E. faecalis MMH594	Resistant to erythromycin and gentamicin	32/ >128
E. faecalis SV587 (VRE)	Resistant to vancomycin	32/ >128
E. faecium E1162	Resistant to ampicillin	32/ >128
E. faecium E0120 (VRE)	Resistant to gentamicin and vancomycin	32/ >128
E. faecium ERV102 (VRE)	Resistant to ampicillin and vancomycin, and displays high levels of resistance to streptomycin.	32/ >128
E. faecium ATCC6569	-	32/ >128
E. faecium ATCC 700221 (VRE)	Resistant to vancomycin and teicoplanin	32/ >128
L. monocytogenes F4244		32/ >128
L. monocytogenes J0161	β-hemolytic, slow rhamnose fermenter	32/ >128
L. monocytogenes ATCC 13932	β-hemolytic	32/ >128
L. monocytogenes ATCC 19112	β-hemolytic	32/ >128
L. monocytogenes ATCC 19111	β-hemolytic	32/ >128
L. monocytogenes ATCC 19114	β-hemolytic	32/ >128
S. pneumoniae 51916	Resistant to cephalosporins	64/ >128
S. pneumoniae 70677	Resistant to erythromycin, penicillin, and tetracycline	64/ >128
Bacillus anthracis	Stern vaccine strain	16/64
B. anthracis UM23	Weybridge strain	16/64
B. anthracis AMES35	Isolated from 14-month-old heifer that died in Texas in 1981.	16/64

Supplementary Figure S1

a)

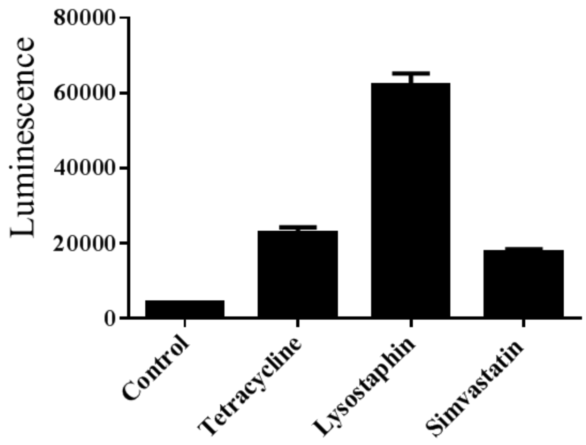

b)

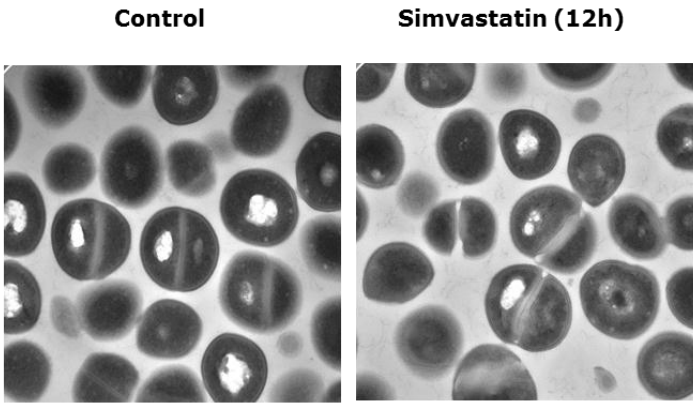

Supplement: Supplementary Information [file srep16407-s1.pdf]
